# Detection of Engineered Copper Nanoparticles in Soil Using Single Particle ICP-MS

**DOI:** 10.3390/ijerph121215020

**Published:** 2015-12-10

**Authors:** Jana Navratilova, Antonia Praetorius, Andreas Gondikas, Willi Fabienke, Frank von der Kammer, Thilo Hofmann

**Affiliations:** 1 Department of Environmental Geosciences, University of Vienna, Althanstrasse 14, 1090 Vienna, Austria; jana.navratilova@univie.ac.at (J.N.); antonia.praetorius@univie.ac.at (A.P.); andreas.gondikas@univie.ac.at (A.G.); willi.fabienke@gmail.com (W.F.); 2 Environmental Sciences Research Network, University of Vienna, 1010 Vienna, Austria

**Keywords:** spICP-MS, soil contamination, copper, nanoparticles

## Abstract

Regulatory efforts rely on nanometrology for the development and implementation of laws regarding the incorporation of engineered nanomaterials (ENMs) into industrial and consumer products. Copper is currently one of the most common metals used in the constantly developing and expanding sector of nanotechnology. The use of copper nanoparticles in products, such as agricultural biocides, cosmetics and paints, is increasing. Copper based ENMs will eventually be released to the environment through the use and disposal of nano-enabled products, however, the detection of copper ENMs in environmental samples is a challenging task. Single particle inductively coupled plasma mass spectroscopy (spICP-MS) has been suggested as a powerful tool for routine nanometrology efforts. In this work, we apply a spICP-MS method for the detection of engineered copper nanomaterials in colloidal extracts from natural soil samples. Overall, copper nanoparticles were successfully detected in the soil colloidal extracts and the importance of dwell time, background removal, and sample dilution for method optimization and recovery maximization is highlighted.

## 1. Introduction

With the rapid development of nanotechnology, consumer products containing engineered nanoparticles (ENPs) are commonly available on the market, and release of ENPs to the environment cannot be avoided. The most frequently used nanoparticles in consumer products are generally metal oxide nanoparticles; namely titanium, zinc, copper and iron (-oxides). Predominant applications are pigments and antibacterial agents [1]. Environmental and safety issues are particularly important for copper-based nanomaterials because of their large-scale use in marine antifouling paints, agriculture biocides, wood preservatives and their high redox activity and toxicity reported from *in vitro* studies. Developed for use as biocides, copper oxide nanoparticles (CuO NPs) have been shown to have the expected toxic effects on aquatic organisms including *Allivibrio fischeri* and *Daphnia magna* [2,3]. With the intended and proven effect on organisms comes the need for investigating the fate of CuO NPs outside of their intended use, once entering different environmental compartments like water, soil and soil exposed organisms. However, until today, reliable methods to detect, identify and quantify engineered CuO NPs in the presence of natural nanoparticles are still missing.

The detection of ENPs in environmental matrices (e.g., soils or sediments) is a challenging task. Most of the methods used for nanoparticle detection are hindered by the low concentrations of ENPs in the environment and comparably high background concentrations of particles composed of the important elements (Ti, Fe, Cu, Zn) in natural waters and soils [4]. Natural background levels of Ti, Fe, Ce and Cu fall in ranges exceeding the expected environmental concentrations of ENPs by orders of magnitude [5,6]. We therefore need to identify analytical handles specific to ENPs (e.g., isotope or elemental ratios, morphology, surface properties) and develop strategies to distinguish between ENPs and natural backgrounds to be able to assess the anthropogenic portion among natural NPs [7,8].

The high natural background of elements making up several of the high-volume metal oxide ENPs and the need to go beyond detection towards identification, quantification and even further characterization of the ENPs (e.g., in terms of particle size, composition, shape, surface chemistry) requires the analysis on an individual particle basis. In this context, time-resolved or single particle ICP-MS (spICP-MS) appears to be a particularly promising technique for nanoparticle analysis. This method has already shown great potential for tracing ENPs (e.g., AgNPs, TiO_2_) in the aquatic environment [4,9], and it is the most promising technique for the routine detection at environmentally relevant concentration levels (ng·L^−1^ and below). The principle of the spICP-MS lies in the different behavior between dissolved and particulate metal in the plasma, thereby making use of the granularity or discontinuity of nanoparticulate dispersions. In a time-resolved mode, dissolved metal ions provide a continuous signal, in contrast, particles appear as pulses above the continuous background [10]. The frequency of the pulses can be related to the particle number concentration if the nebulization efficiency is known and the intensity of each pulse is proportional to the mass of the nanoparticle. If information about particle geometry, stoichiometry and density is available, then the mass of the analyte can be converted to the particle size [11]. In the case of natural particles, however, where information about total element composition, morphology and density is missing, it is not possible to accurately size the particles; the main focus of the spICP-MS analysis is placed on counting particle occurrences. 

**Figure 1:**
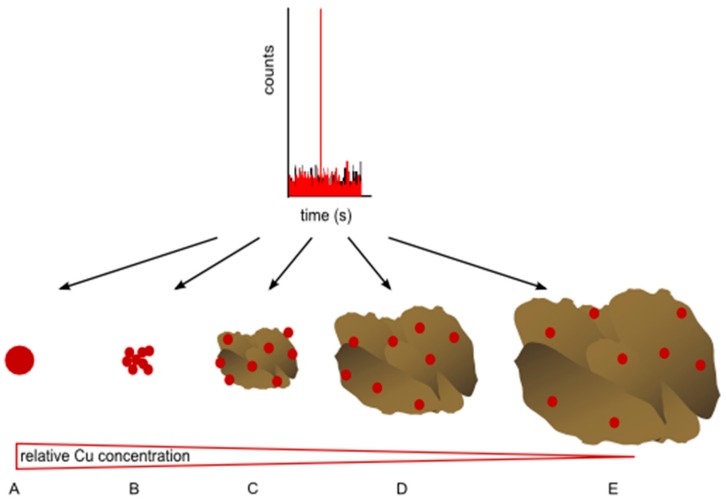
Schematic representation of the possible origins of a given single particle signal in the ICP-MS for a specific element. The intensity of the spike corresponds to a fixed mass of the analyzed element, which can be present in the sample as a single (nano-) particle (**A**), a particle aggregate (**B**) or as the given element distributed within a (natural) particle composed of a variety of elements (**C**–**E**). Note that with decreasing relative concentration of the analyzed element in the particle, the total size of the analyzed particle increases.

When typical quadrupole ICP-MS instruments are operated in the time-resolved single particle mode, the mass spectrometer must be locked on one isotope and thereby loses the multi-element capability for which the ICP-MS technique has become prominent. This complicates the detection of analytical interferences on the target isotope and prevents the application of isotope or elemental ratio analysis on a single particle level [7,12]. Another problem arises from the fact that the smallest detectable particle size depends (among many other factors) on the mass fraction of the selected element in the measured particle. The consequence is that particles made up of many different elements must have a much larger size to be detected than those composed of one element as demonstrated in Figure 1. This appears as a further critical disadvantage, but can be utilized to distinguish certain background and engineered nanoparticles in natural samples.

Here, we demonstrate the application of spICP-MS for the detection of engineered CuO NPs in soil matrices against a natural copper background. Copper is present as a trace element in primary minerals in the form of copper sulfide inclusions and isomorphic substitutes for Fe, Mg, Ca, K, or Na in some silicates [13]. In oxic soils, copper can be predominantly associated with soil organic matter (SOM); under fluctuating redox conditions, SOM associated copper may undergo mineralization or biomineralization to form copper sulfide and to a lesser extent even metallic copper [14,15,16]. Organic substances strongly influence the speciation of copper in water and soil. Soil humic acid forms complexes with Cu(I) and Cu(II), and promotes the oxidation of Cu(0) to Cu(I) [17,18]. We therefore hypothesize that naturally occuring copper will in almost all cases be distributed rather homogeneously throughout the soil matrix and not appear as individual particulate assemblies, with the necessary size and high relative Cu concentration to exhibit a particle spike in spICP-MS measurements (which has been observed for oxide-forming elements such as titanium and cerium). The median concentration for copper in European topsoil and sediments are 12.9 and 17 mg·kg^−1^, respectively [19] and can reach several 100 mg·kg^−1^ in contaminated areas. However, as long as the natural copper background is distributed between different phases and does not appear as individual particles or in local particulate enrichments, the particle size of such a hypothetical natural copper-carrying particle must be in the >0.5 µm size range to become detectable as a Cu-spike in spICP-MS. For example, a natural particle with 15 mg·kg^−1^ copper would have to be present at a size of about 3.5 µm to generate a particle signal equivalent to a 70 nm CuO NP (see Section S1 in the Supporting Information and Figure 1 for illustration). Conversely, a natural particle of 0.5 µm or smaller would have to contain at least 5 g·kg^−1^ of copper to result in an equivalent signal. For this reason, we assume that conventional one-element spICP-MS can be applied to detect the presence of engineered CuO NPs in most natural waters and soils.

In this study, we conducted experiments to prove this hyphothesis on different natural soil matrices, namely an aquifer colloidal extract and three topsoil colloidal extracts. A colloidal extract is the resulting stable dispersion of colloids and nanoparticles extracted from solid matrices such as soils or sediments. The aqueous extraction procedure involves steps to release and de-agglomerate colloids and nanoparticles by physical and chemical means, an upper size cut-off between 0.1 and 1 µm by centrifugation and measures to stabilize extracted particles to prevent agglomeration in the extracts. All samples are first thoroughly characterized and screened with spICP-MS to confirm the absence of detectable particulate copper in the natural samples. Then, the samples are spiked with engineered CuO NPs and spICP-MS is used to detect the CuO NPs contamination to demonstrate the applicability of the new method. Three parameters, namely dilution factor, dwell time, and false positive identification, are discussed in the context of environmental nanometrology.

## 2. Experimental Section

### 2.1. Materials

Ultrapure water (Milli-Q) was obtained from a Millipore element system (Millipore, Milford, MA, USA) and used throughout the work. Nitric acid (67%–69%) of PlasmaPURE *Plus* quality, which was used to prepare calibration standards, was obtained from SCP Science (Quebec, Canada). A certified copper reference standard solution of 1000 µg·mL^−1^ in 2%–3% nitric acid was obtained from Merck (Darmstadt, Germany). As a quality control, Standard Reference Material 1640a Trace Elements in Natural Water (NIST, USA) was used.

### 2.2. Copper Oxide Nanoparticles

The CuO nanopowder of spherical morphology was purchased from PlasmaChem and dispersed at a concentration of 50 mg·L^−1^ in Milli-Q water using a sonication bath. Zeta potential measurements were performed using a Zetasizer Nano ZS instrument (Malvern Instruments, UK). The zeta potential value was obtained from three consecutive measurements. The particle size of the dispersible fraction was measured by dynamic light scattering (DLS) (scattering angle 173°) in triplicates.

### 2.3. Natural Samples

#### 2.3.1. Saturated Groundwater Aquifer Colloid Extract 

A stable colloid extract from the matrix of a saturated groundwater aquifer (SG) was used. The extract with a particle concentration of 120 mg·L^−1^ and an estimated average particle density of 2.6 g·cm^−3^ contains particles with average particle size of 100 nm as determined by Field Flow Fractionation. Assuming the natural particles are spherical, their theoretical number concentration is 1.27 × 10^11^ mL^−1^. A more detailed description and characterization of the sample can be found elsewhere [20,21]. 

#### 2.3.2. Topsoil Colloid Extracts: Sampling Area

Topsoil samples used in this study originate from two large city parks in Vienna (Austria), the Lainzer Tiergarten (sample LT1) and the Plötzleinsdorfer Schlosspark (samples PS2 and PS3). The specific sample locations were chosen with a minimum distance of 200 m from the next road and far from agricultural activities to avoid potential contamination by anthropogenic Cu or CuO NPs. The subsamples of each location were collected with a minimum distance of 30 m from each other and each subsample is a composite sample mixed from three equal parts sampled within a 2 m radius. The first 1–3 cm of the specific sampling sites were removed due to high organic debris content, and soil samples were taken at a depth of 3–10 cm. The composite soil samples were stored in the dark at 4 °C.

#### 2.3.3. Topsoil Colloid Extracts: Colloid Extraction Procedure

The soil samples were wet-sieved to <32 µm with Milli-Q water (~100–300 mL of Mili-Q for 100 g of soil), frozen and freeze-dried. An aqueous extraction method, adapted from Plathe *et al.* (2010) and von der Kammer (2004) [21,22], was used to obtain colloidal suspensions from the <32 µm soil fractions. First, exchangeable divalent cations were removed by mixing 4 g of freeze-dried sample with 40 mL of 0.1 M NaCl in 50 mL centrifuge vials, which were placed on a shaker at 200 rpm for 21 h in the dark. The mixtures were then centrifuged at 2.490× *g* for 51 min to give a 100 nm size cut-off for particles (assuming an average density of 2.6 g·cm^−3^) (see Equation (1) in [21]). At 0.1 M NaCl, all particles were expected to be aggregated to sizes larger than 100 nm and removal of all particles from the supernatant was inspected visually using a standard red laser pointer, which revealed no scattered light visible to the naked eye at a scattering angle of ~30°. The supernatant was discarded and the NaCl was washed out to reverse the aggregation by adding 35 mL of Milli-Q water and the sediments were resuspended by 30 s of vortexing, 10 min of sonic bath and 24 h of shaking. The samples were centrifuged to a 300 nm cut-off (1.507× *g*, 5 min 40 s). The top 20 mL of the supernatant were carefully collected with a pipette and replaced by 20 mL of Milli-Q water. 16 more washing steps (5 min sonic bath, 30 s vortexing, minimum 1 h shaking, 10 min sonic bath, centrifugation to 300 nm cut-off) were performed, collecting and replacing 20 mL of supernatant after each centrifugation step, until the supernatants returned substantially less turbid. All supernatant fractions (colloidal extracts) collected for each sample were combined and stored in the dark at 4 °C. 

#### 2.3.4. Total Copper Measurement

The total copper content was determined in all colloidal extracts (SG, LT1, PS2, PS3) with an inductively coupled plasma mass spectrometer (ICP-MS) (7700 series ICP-MS, Agilent Technologies, Santa Clara, CA) after microwave digestion (Multiwave 3000, PerkinElmer, MA, USA). For the digestion, a mixture of concentrated HNO_3_/HCl/H_2_O_2_ was added to 10 mL of extract. The microwave power was set to reach 800 W within 15 min and held constant for 10 min. Samples were then cooled to room temperature, diluted to 50 mL or 25 mL using Milli-Q water prior to analysis with ICP-MS.

#### 2.3.5. Spiking Experiments

The soil colloidal extracts (LT1, PS2, PS3) were placed in a sonic bath for 10 min before dilution with Milli-Q water (1:100 dilution). The SG sample contains less suspended solids and was therefore diluted by a factor of 1:10. All colloidal extracts (SG, LT1, PS2, PS3) were spiked with the dispersible fraction of the CuO NP suspension and manually shaken for 1 min prior to spICP-MS analysis. 

### 2.4. Single Particle ICP-MS

#### 2.4.1. Instrument Settings

The measurement was performed using an Agilent 7900 ICP-MS (Agilent Technologies, Santa Clara, CA). For the analysis of copper isotope 63 was chosen because of its high abundance (69%), which provides the highest instrumental response. Helium as a collision gas at a flow rate of 4.5 mL/min was used in order to remove the polyatomic ^40^Ar^23^Na interference on isotope ^63^Cu, since sodium is commonly found in soils at high concentration levels and is listed as an impurity by the nanoparticle provider. Before analysis the instrument was manually tuned using 10 µg·L^−1^ ionic copper standard solution for the highest instrumental response. As a quality control, Standard Reference Material 1640a Trace Elements in Natural Water (NIST, USA) was analyzed for copper content to verify the method accuracy. The 5 ms dwell time was chosen since it was demonstrated in a previous study on silver nanoparticles that using a 5 ms dwell time provides the most accurate results in terms of particle counting [23,24,25]. To allow for peak profiling and background reduction the dwell time was reduced to 0.1 ms. The instrumental settings used for the single particle analysis are summarized in Table S1 (Supporting Information). 

It is important to emphasize that spICP-MS is a rapid and sensitive method providing information only about particle mass and particle number. However no information about particle shape is available from this technique and care should be taken when comparing with other size measuring techniques. All sizing attempts rely on shape and density assumptions, which can, especially in the case of natural particles, result in significant errors.

To relate the number of detected single particles to the particle concentration in the analyzed suspension, the nebulization efficiency was measured in triplicates using a well characterized gold 60 nm particulate standard (BBI, UK). Sample flow rate was measured with a TruFlo flow meter (GlassExpansion, Australia) and the NP concentration, the size and the density of the gold nanoparticles provided the theoretical number of aspirated particles per unit time. The number of particles reaching the plasma was determined by the frequency of detected events, and the ratio of the two numbers was used to determine the nebulization efficiency. 

#### 2.4.2. Data Analysis

The spICP-MS data processing was done using Matlab R2012b and OriginPro 9.1. The main challenge in the processing of this spICP-MS data was the discrimination between the background, dissolved, and particulate signal. Setting an exact threshold between particle and background is not a trivial task. Here, we apply an iterative approach as described in literature to extract the mean intensity, $\mu_{\text{diss}}$, and standard deviation, $\sigma_{\text{diss}}$, of the dissolved Cu signal from the raw data [23,24,25]. To separate background and particle events, we set a particle detection threshold of 7$\sigma_{\text{diss}}$. Only data above $\mu_{\text{diss}}$ + 7$\sigma_{\text{diss}}$, are considered as particle events, whereas the data below this threshold are removed. This comparatively high threshold of 7$\sigma_{\text{diss}}$ was chosen to minimize the contribution of dissolved signal to the particle counts. Similarly high thresholds have been applied before in studies dealing with high dissolved backgrounds [25].

For the data collected at higher time resolution (dwell time of 0.1 ms) it has to be taken into account that one particle event is often split over several acquisition time intervals. In this case, the particle signals occurring over multiple time points were integrated to obtain actual particle numbers and signal intensities. 

A further challenge in counting particles in spICP-MS is the possible presence of false positive signals, from machine noise or carry-over of particles trapped in the system, which might significantly increase the total particle count for samples of low particle number concentration. In order to reliably report detected particle numbers, we define a confidence threshold for detected particle numbers represented by the mean number of false positives detected in several blank measurements (Milli-Q) plus three times its standard deviation and we subtract this value from the particles detected in each sample. 

## 3. Results and Discussion

### 3.1. Characteristics of CuO NPs

The CuO NP dispersion in Milli-Q resulted in fast particle settling, indicating a strong tendency of the NPs to agglomerate; therefore, only the dispersible fraction was further characterized and used for the spiking experiments. The dispersed CuO fraction was characterized by spICP-MS and DLS. The hydrodynamic diameter observed by DLS measurement was 208 ± 7 nm, with a polydispersivity index (PDI) of 0.39 and the surface charge (as Zeta potential) was determined according to +30.5 ± 2.8 mV. The particle number concentration in the suspension was 14.2 ± 3.1 × 103 mL−1 (248 ± 54 particles detected within the 60 s of measurement using 5 ms dwell time and after the $7\sigma_{\text{diss}}$ cut off). Based on the spICP-MS data the suspension of pure CuO NPs contains mainly particles in size range 35–60 nm (Figure S1, Supporting Information), which were not detected by the DLS analysis due to the obtained intensity weighted distribution by DLS which highly overemphasizes large particles (I ~ r^6^). The background equivalent size (size detection limit calculated from the lowest detectable Cu calibration standard of 20 ng·L^−1^) for CuO nanoparticles of spherical shape and density 6.5 g·cm^−3^ was 15 ± 10 nm. The nebulization efficiency for the Au nanoparticulate standard was 5.2 ± 1.1 %. The accuracy of the measurement was tested by the analysis of the SRM 1640a, which has a certified copper concentration of 85.75 ± 0.51 µ·L^−1^. Here, a value of 84.16 ± 0.89 µ·L^−1^ (*n* = 3) was obtained.

### 3.2. Characteristics of Soil Colloidal Extracts

#### 3.2.1. Elemental Composition

The multi element analysis of the colloidal extracts by ICP-MS after microwave digestion (Table 1) showed high concentrations (in the mg·L^−1^ range) of Fe, Al and Si, which are commonly found in the form of oxides and clay minerals in natural soils. The total copper content was in a much lower concentration range (µg·L^−^^1^). The pH of the aqueous colloidal suspensions ranged from 6.6 to 7.2 (Table 1).

**Table 1:** Elemental composition and pH of the colloidal extracts.

Sample Code	Si	Al	Fe	Mg	Mn	Ni	Zn	Cu	pH
	mg·L^−1^	mg·L^−1^	mg·L^−1^	mg·L^−1^	mg·L^−1^	µg·L^−1^	µg·L^−1^	µg·L^−1^	
SG	27.1	10	19.2	0.9	5.2	22.2	82.4	30	7.0
LT1	334	644	130	29.8	1.1	139.9	613.7	102	6.8
PS2	808	427	161	42.5	2.8	205.2	672.0	179	7.2
PS3	668	371	190	39.1	1.6	224.2	743.2	217	6.6

#### 3.2.2. Single Particle ICP-MS: Optimizing Sample Dilution 

Optimizing the dilution factor is a critical step in spICP-MS analysis, when using a time resolution in the millisecond range (5–10 ms), especially when analyzing unknown samples. Increasing the dilution factor reduces the chance of coincidences (more than one particle enters the plasma in the same acquisition time interval) and also improves the size detection limit, by decreasing the background signal and signal variation of dissolved species, in which the spikes of small particles may be lost. When the dilution factor is too high, extended measurement times are required to obtain sufficient particle events, which increase the chance of false positive signals, measurement time and costs of analysis. Several strategies can be applied in order to reduce the background other than simple dilution of the sample. If the particle number in solution is too low and further dilution is not possible, the dissolved species can be removed using ion exchange column or (cross-flow-) filtration. However, possible interactions with the column resin or filter leading to the loss of particles have been reported [26]. Another strategy to reduce background signals without any sample manipulation consists of simply shortening the dwell time. The effect of dwell time on the background is also demonstrated in this work. 

As can be seen from Table 1, the concentration of Cu is several orders of magnitude lower than major elements such as Si, Al, and Fe in the aquifer extract (SG) as well as in the topsoil samples. Some elements are likely to form solid phases in the range of micrometers, or larger (e.g., Ca, Mg) and other elements, such as Fe, are mostly present as nanoparticles or represent a part of nanoparticles in natural samples and can therefore be used as surrogates of the nanoparticulate fraction in natural samples [27]. We used the spICP-MS method with a slight modification (H_2_ gas instead of He) to detect iron-bearing nanoparticles and acquire an estimation of the abundance of natural nanoparticles in our system. Indeed, as shown in Figure S2, Fe-bearing nanoparticles were detected in large numbers. When performing the same spICP-MS analysis to screen for Cu-containing natural NPs, none were detected. Due to the lower concentration of copper (0.03 mg·L^−1^) compared to iron (19.2 mg·L^−1^), the same dilution scheme cannot be applied. The aquifer extract was therefore measured undiluted, in order to improve the counting of especially the larger particles that are expected to be present at lower numbers. Measuring without dilution, however, resulted in higher background signal intensity and signal variation, which may overwhelm the signal of smaller particles and render their detection impossible. It becomes apparent that dilution of the sample is not only a critical point for preventing doublets to be counted as one particle (at higher sample concentrations) or poor counting statistics (at low sample concentrations) but is also pivotal for identification of a certain type of NP in the sample, especially when it is present with a broad size distribution. At low sample concentrations, the dissolved background is low and smaller particles (~25 nm) become detectable. Small particles might appear in sufficient particle numbers even under high sample dilution, because at constant particle mass concentrations for all sizes, they are present at much higher numbers compared to the larger particles (>100 nm). However, at these low sample concentrations, larger particles, each carrying a much higher per-particle mass and appearing at much lower number concentrations, might not show up at all during a normal measurement duration of 60 s. At higher sample concentrations, the number of large particles can be sufficient, but due to the higher dissolved background, the smaller particles are not detected because they cannot be separated from the background signal.

To ensure that no natural Cu-NPs are overlooked as a result of inappropriate dilution, the SG sample was serially diluted prior to the spICP-MS analysis (Figure S3, Supporting Information) down to a total copper concentration of 3 ng·L^−1^. Such low background concentration allows for the detection of small particles, close to the theoretical detection limit of 15 nm but would require extensive measurement times to detect low number concentrations. A final dilution of 1:10 for sample SG was chosen for the subsequent analyses as an optimal balance between low background signal intensity and low numbers of large Cu-containing particles. For the topsoil colloidal extracts (LT1, PS2, PS3), with concentrations of Cu of about one order of magnitude higher than SG (Table 1), a dilution of 1:100 was chosen accordingly.

The data in Table 2 shows that the particle signals for Cu detected in all natural samples falls in the same order of magnitude as the number of false positive signals recorded in the blanks (Milli-Q water). As hypothesized, particulate copper is not detectable in the colloidal soil extracts at any dilution step. The spICP-MS data exhibited a constant signal above the blank level, indicating that the vast majority of copper is truly dissolved and that the natural particles do not contain a sufficiently high proportion of Cu to result in particulate signals, which may be explained by the presence of SOM associated copper complexes [14].

**Table 2:** Number of particle spikes, particle number concentration (mL^−1^) at 5 ms and 0.1 ms dwell time for the CuO NP suspension, unspiked and spiked soil extracts. Additionally, the average data for the blank measurements (in Milli-Q) are provided (for 5 ms n_blanks_ = 13 and for 0.1 ms n_blanks_ = 8).

Sample	5 ms				0.1 ms			
	NP Spikes	[NP]	µ_diss_	7σ_diss_	NP Spikes	[NP]	µ_diss_	7σ_diss_
	min^−1^	mL^−1^	(counts)	(counts)	min^−1^	mL^−1^	(counts)	(counts)
**Blank (Milli-Q)**	0.9 (±1.0)	53 (±59)	2.3 (±0.5)	9 (±1.8)	21 (±21)	1.2 (±1.2) × 10^3^	1.1 (±0.01)	2 (±0.1)
**CuO NPs**	286	1.6 × 10^4^	10	63	816	4.7 × 10^4^	1	5
**SG (unspiked)**	1	5.7 × 10^1^	577	220	4	2.3 × 10^2^	12	26
**LT1 (unspiked)**	8	4.6 × 10^2^	221	135	60	3.4 × 10^3^	4	15
**PS2 (unspiked)**	3	1.7 × 10^2^	431	205	30	1.7 × 10^3^	8	22
**PS3 (unspiked)**	2	1.1 × 10^2^	460	202	20	1.1 × 10^3^	9	23
**SG + CuO NPs**	20	1.1 × 10^3^	582	245	30	1.7 × 10^3^	12	26
**LT1 + CuO NPs**	63	3.6 × 10^3^	224	169	192	1.1 × 10^4^	5	16
**PS2 + CuO NPs**	32	1.8 × 10^3^	528	261	156	8.9 × 10^3^	8	22
**PS3 + CuO NPs**	42	2.4 × 10^3^	442	229	106	6.1 × 10^3^	9	23

### 3.3. Detection of CuO NPs in Spiked Colloidal Extracts

**Figure 2:**
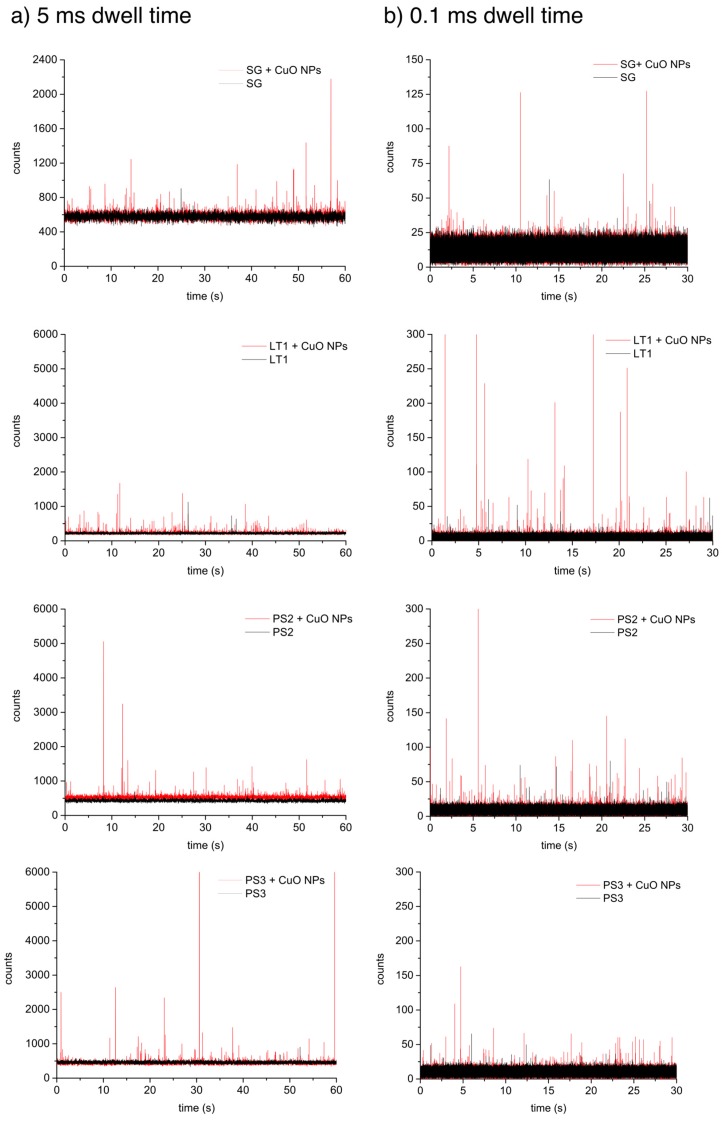
Raw spICP-MS data of the natural colloidal extracts (black line) and colloidal extracts spiked with engineered nanoparticles (red line) at a dwell time of 5 ms (**a**) and 0.1 ms (**b**).

All colloidal extracts were spiked with the same amount of the CuO NP suspension (Cu concentration of 0.1 µg·L^−1^) and analyzed by spICP-MS. In Figure 2, the raw spICP-MS data using 5 and 0.1 ms dwell times are shown for all samples spiked with CuO NPs. 

As can be seen in the Figure 2, the addition of CuO NPs to the samples is clearly detectable and visible as spikes above the constant background in the spICP-MS analysis runs. In Figure 2a with data acquisition intervals of 5 ms, the background signal remains high for all runs, especially those of SG. As pointed out earlier, this elevated background level could mask a fraction of small particles, and one option to counter this effect is to reduce the data acquisition intervals (dwell time). With data acquisition times above 1 ms, the NP measurement is based on event monitoring and the number of occupied time slots and the peak height is used for the construction of number concentrations and particle size. With acquisition times below 1 ms (preferred ≤100 µs) the single event is spread across several time slots and data treatment has to take this into account. The dwell time was lowered to 0.1 ms as shown in Figure 2b to allow small particles to become detectable. Increasing the time resolution to 0.1 ms decreased the background by two orders of magnitude. The effect of the dwell time on particle number concentration is summarized in Table 2. To achieve a high discrimination of particles from the dissolved background a 7$\sigma_{\text{diss}}$ threshold was applied for all samples. In this study, a strong emphasis is placed on avoiding falsely assigning natural background signal towards the engineered CuO NPs count. Therefore, a rather conservative threshold of 7$\sigma_{\text{diss}}$ was applied to all samples, but the effects of using higher and lower thresholds are also reported in Tables S2 and S3 in the Supporting Information.

As shown in Table 2, a low frequency of false positive spikes was also observed in the blanks (Milli-Q water). Therefore, a confidence threshold was defined (only particles above the mean number of false positives plus three times its standard deviation) and the NP spikes were evaluated based on this criterion (Table 2). The final reported particle numbers are summarized in Table 3 and represent the number of Cu-containing NPs we can confidently consider as true particle events.

Interestingly, the application of the confidence threshold led to particle number concentrations falling below the particle detection limit (230 mL**^−^**^1^ for 5 ms and 4.8 × 10^3^ mL**^−^**^1^ for 0.1 ms) like in the case of sample SG that was artificially spiked with the CuO NP suspension and analyzed with 0.1 ms acquisition. This is mainly explained by the comparatively low number of Cu-NPs detected in SG due to the particularly highly dissolved background present in this sample, masking a large proportion of the added CuO NPs.

**Figure 3:**
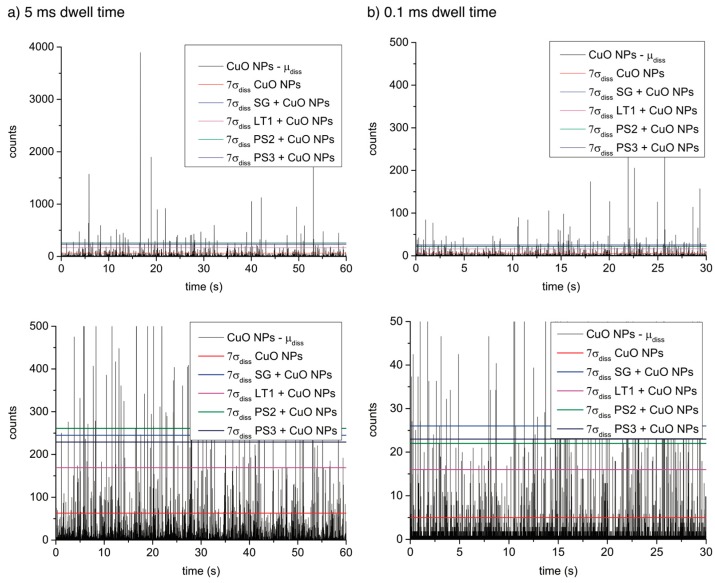
Raw spICP-MS data of the engineered CuO NPs (with the mean dissolved background subtracted) compared to the level of 7$\sigma_{diss}$ of the different samples at a dwell time of 5 ms (**a**) and 0.1 ms (**b**).

The maximum recovery (calculated by comparing the detected number concentration of CuO NPs in the spiked samples to the number concentration in the pure CuO NPs suspension, after applying the confidence threshold) was highest for sample LT1 (21% at 5 ms and 15% at 0.1 ms) and lowest for SG (6% and BCT, respectively), which can be explained by LT1 having the lowest and SG the highest thresholds for particle detection (Table 2, Figure 3) due to the background of dissolved copper. 

**Table 3:** NP concentration (mL**^−^**^1^) corrected by subtracting the concentration of false positives (blank) plus three times its standard deviation. CT confidence threshold, BCT = below confidence threshold.

Sample	5 ms		0.1 ms	
	NPs above CT	[NP] above CT	NPs above CT	[NP] above CT
		mL^−^^1^		mL^−^^1^
CuO NPs	Yes	1.6 × 10^4^	Yes	4.2 × 10^4^
SG (unspiked)	No	BDL	No	BCT
LT1 (unspiked)	Yes	2.3 × 10^2^	No	BCT
PS2 (unspiked)	No	BCT	No	BCT
PS3 (unspiked)	No	BCT	No	BCT
SG + CuO NPs	Yes	9.1 × 10^2^	No	BCT
LT1 + CuO NPs	Yes	3.4 × 10^3^	Yes	6.2 × 10^3^
PS2 + CuO NPs	Yes	1.6 × 10^3^	Yes	4.1 × 10^3^
PS3 + CuO NPs	Yes	2.2 × 10^3^	Yes	1.3 × 10^3^

According to the spICP-MS data, the suspension of pure CuO NPs Figure S2 (Supporting Information) contains a large fraction of particles with a mean size of 43 nm (5 ms), which was not visible by DLS analysis. Small particles are masked by the highly dissolved background in the natural samples (Figure 3) and only comparatively large CuO NPs can be detected in those natural soil extracts. The 7$\sigma_{diss}$ threshold for the extracts is equal to particle size of 99–117 nm for the 5 ms dwell, while using 0.1 ms, the particle size is reduced to 48–57 nm, close to the important size range for the pure CuO-NP dispersion. Only particles above the size thresholds can be reliably detected in either case. 

## 4. Conclusions 

We present a method for detecting engineered CuO NPs in natural soil extracts using spICP-MS. For the tested soil samples, spICP-MS analysis of the colloidal extracts reveals that copper is not appearing as detectable natural nanoparticles in the size ranges accessible by the method. This suggests that copper is either adsorbed to natural particles in amounts that do not produce a detectable particle spike and/or as soluble Cu- complexes, most likely with dissolved SOM. This finding offers a useful and straighforward approach for detecting anthropogenic Cu-containing nanoparticles in complex environmental matrices like soil, sediment extracts and others. In this study, a particular emphasis was placed on adjusting sample dilution and dwell times to carefully distinguish CuO NPs against high backgrounds of natural NPs. Overall, we choose a rather conservative approach for particle detection with the goal of avoiding falsely identifying background signal as engineered CuO NP particles. In this context, the need for comparing and correcting detected particles spikes against the presence of false positive spikes is also discussed.

We distinctly detect engineered CuO NPs in all four spiked natural samples. However, the size-based detection limit depends on the level of natural dissolved Cu in the different samples. High Cu background hinders the detection of very small engineered CuO NPs, but larger CuO NPs or agglomerates of smaller NPs can be reliably detected with this method. 

The applied concept to detect engineered copper particles in soil colloidal extracts is based on the assumption that most of the copper is bound to SOM or adsorbed to natural NPs. In this work, we investigated the copper origin in top soil samples, where the toxic environment is favoring SOM-bound and adsorbed copper species. The same scenario might not be applicable in sulfate-reducing soils and sediments where the presence of solid copper in forms of Cu_x_S and Cu^0^ can be expected and the spikes of naturally occurring Cu-containing NPs might mask the detection of added engineered CuO NPs during the spICP-MS analysis. This demonstrates the need for investigations into the nature of natural NPs under various environmental conditions.

The distinction of particulate *versus* dissolved signals is a common issue in the application of spICP-MS to soluble NPs such as Ag NPs, or NPs with a high natural dissolved background, such as Cu in the present case. Currently, research efforts are focused on improving data acquisition modes and data analysis techniques to enable to distinguish particulate and dissolved signals more reliably. We are therefore confident that the method presented here can be improved in the future, to detect smaller NPs despite high Cu backgrounds in soils. Further method development will also include the detection of engineered CuO NPs added directly to bulk soil samples or in test fields to simulate more realistic exposure and sample preparation scenarios.

## Supplementary Files

Supplementary File 1
