# Supplementary material for: Detection of Engineered Copper Nanoparticles in Soil Using Single Particle ICP-MS
Source: Int J Environ Res Public Health. 2015 Dec 10;12(12):15756–68. doi: 10.3390/ijerph121215020 (PMC4690956; doi:10.3390/ijerph121215020)
Supplement: Supplementary File 1 [file ijerph-12-15020-s001.pdf]

Detection of Engineered Copper Nanoparticles in Soil Using Single Particle ICP-MS

Section S1. spICP-MS Signal of Natural Cu-Containing Particles—Theoretical Considerations

Assuming a spICP-MS detection limit of 1 fg Cu and an average density of the natural particle of 2.6 g·cm^{-3} it is possible to calculate the required size of a natural Cu-containing particle with a given average Cu concentration to provide a detectable particle signal in the spICP-MS. A detection limit of 1 fg Cu corresponds to a minimum detectable CuO NP (pure) of about 70 nm. For a natural particle containing on average 15 mg·kg^{-1} Cu (which falls in the range of natural Cu background concentrations for European soils) this means that a particle size of about 3.5 µm is required to provide an equivalent signal.

Conversely, if we wanted to detect a much smaller natural Cu-containing particle by spICP-MS, it would require a much higher Cu concentration: a 0.5 µm particle, for example, would have to contain 5 g·kg^{-1} in order to provide a detectable particle signal.

Section S2. Instrument Settings

Table S1. Optimized instrumental setting (Agilent 7900) for the detection of Cu in single particle mode.

**RF power**	**1550 W**
Plasma gas flow rate	15 L·min^{-1}
Carrier gas flow rate	0.98 mL·min^{-1}
Makeup gas flow rate	0.19 mL·min^{-1}
Nebulizer	Micromist
Isotope monitored	^{63}Cu
Integration time	5 or 0.1 ms
Sample flow rate	390 µL·min^{-1}
Acquisition time	30 s or 60 s
Cell gas/flow rate	He/4.5 mL·min^{-1}

Section S3. Characteristics of CuO NPs

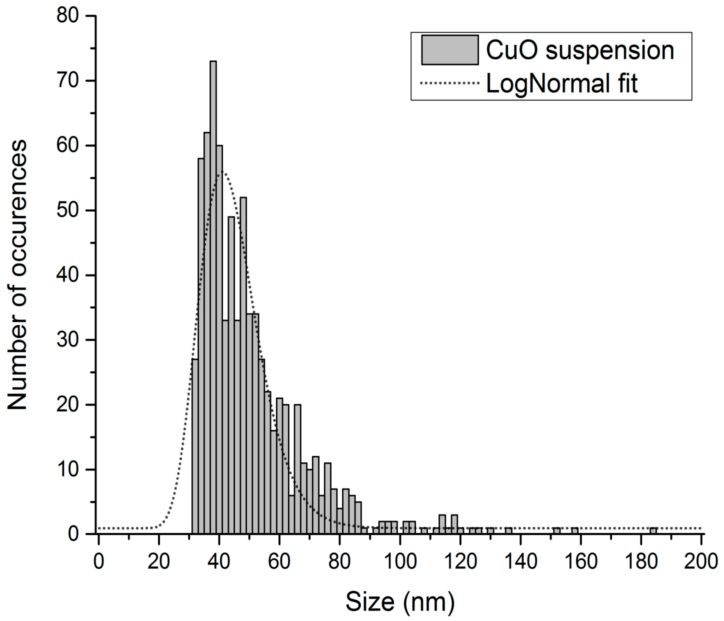

Figure S1. Size distribution of the CuO NP dispersion calculated from single particle data acquired at 5 ms dwell time.

Section S4. Optimizing Sample Dilution

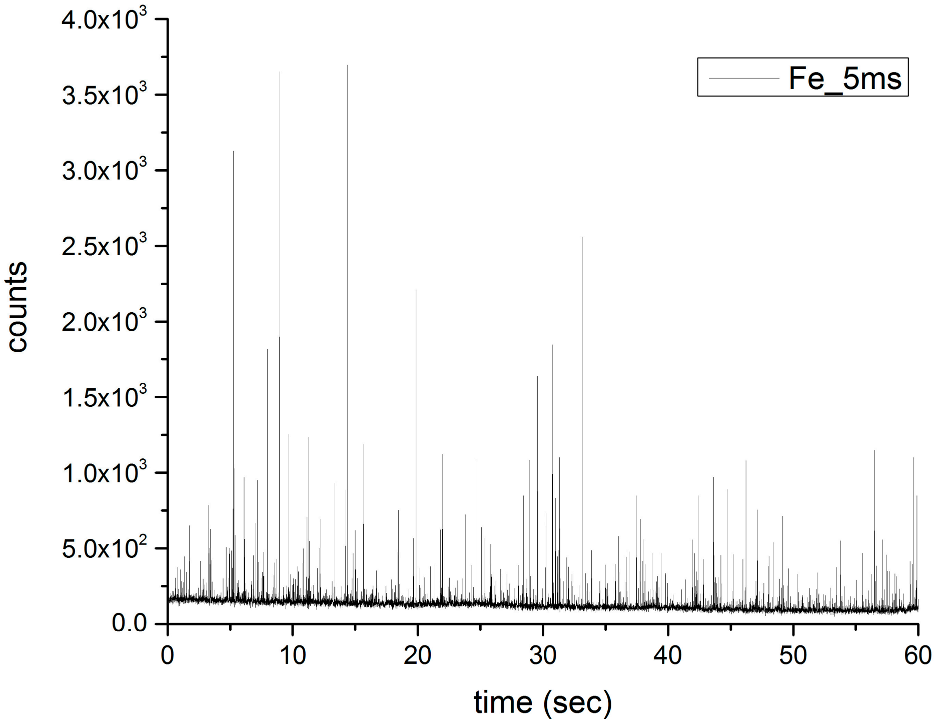

Figure S2. spICP-MS Fe counts of the aquifer extract (SG), diluted by a factor of 20×10^6, providing a particle concentration of 13.4×10^6 L^{-1} using a threshold of 5 sigma.

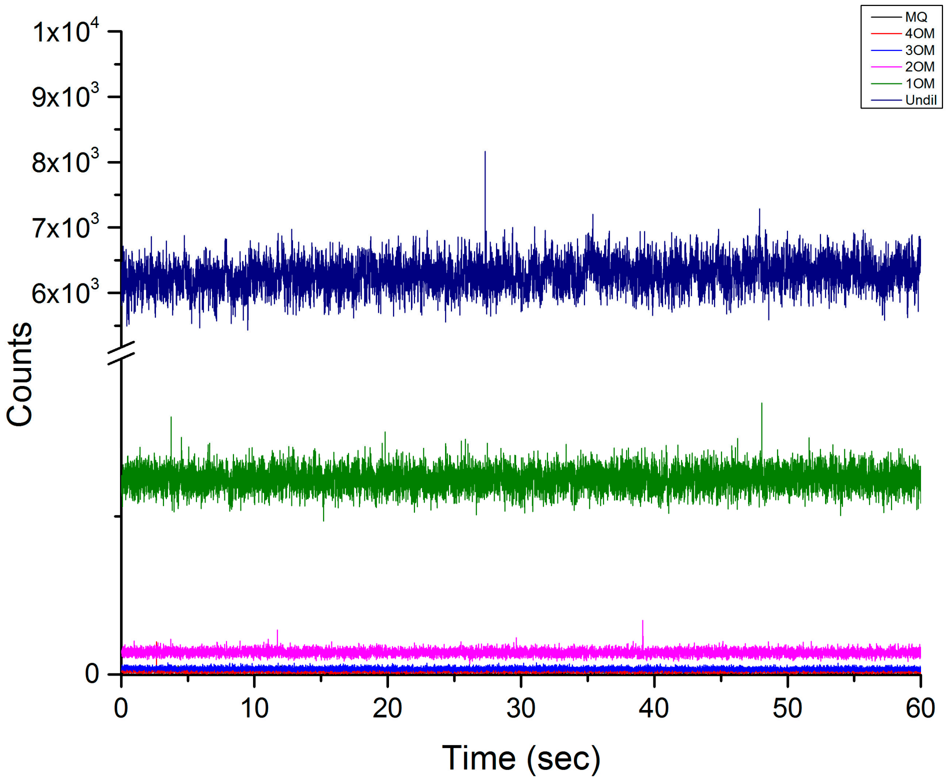

Figure S3. spICP-MS response for Cu to different dilution steps of the aquifer extract (SG) at 5 ms dwell time. "OM" stands for Orders of Magnitude dilution level.

Section S5. Effects of threshold on particle detection

Table S2. Number of particle spikes per minutes and particle number concentration (mL^{-1}) at 5 ms dwell time for all samples as a function of particle detection threshold.

5 ms Dwell Time	$5\sigma_{diss}$		$6\sigma_{diss}$		$7\sigma_{diss}$		$8\sigma_{diss}$	
Sample	NP Spikes min^{-1}	[NP] mL^{-1}	NP Spikes min^{-1}	[NP] mL^{-1}	NP Spikes min^{-1}	[NP] mL^{-1}	NP Spikes min^{-1}	[NP] mL^{-1}
CuO NPs	597	34114	396	22629	286	16343	177	10,114
SG (unspiked)	1	57	1	57	1	57	1	57
LT1 (unspiked)	15	857	11	629	8	457	5	286
PS2 (unspiked)	7	400	5	286	3	171	2	114
PS3 (unspiked)	3	171	2	114	2	114	1	57
SG + CuO NPs	41	2343	28	1600	20	1143	17	971
LT1 + CuO NPs	128	7314	88	5029	63	3600	56	3200
PS2 + CuO NPs	60	3429	41	2343	32	1829	28	1600
PS3 + CuO NPs	77	4400	61	3486	42	2400	37	2114

Table S3. Number of particle spikes per minutes and particle number concentration (mL^{-1}) at 0.1 ms dwell time for all samples as a function of particle detection threshold.

**0.1 ms Dwell Time**	**5σ$_{diss}$**		**6σ$_{diss}$**		**7σ$_{diss}$**		**8σ$_{diss}$**	
**Sample**	**NP Spikes min^{-1}**	**[NP] mL^{-1}**	**NP Spikes min^{-1}**	**[NP] mL^{-1}**	**NP Spikes min^{-1}**	**[NP] mL^{-1}**	**NP Spikes min^{-1}**	**[NP] mL^{-1}**
**CuO NPs**	1794	102514	1270	72571	816	46629	710	40571
**SG (unspiked)**	16	914	6	343	4	229	4	229
**LT1 (unspiked)**	156	8914	76	4343	60	3429	40	2286
**PS2 (unspiked)**	104	5943	58	3314	30	1714	24	1371
**PS3 (unspiked)**	74	4229	38	2171	20	1143	10	571
**SG + CuO NPs**	102	5829	48	2743	30	1714	24	1371
**LT1 + CuO NPs**	414	23657	280	16000	192	10971	144	8229
**PS2 + CuO NPs**	362	20686	214	12229	156	8914	124	7086
**PS3 + CuO NPs**	252	14400	166	9486	106	6057	86	4914

© 2015 by the authors; licensee MDPI, Basel, Switzerland. This article is an open access article distributed under the terms and conditions of the Creative Commons Attribution license (http://creativecommons.org/licenses/by/4.0/).
